# The association between concealing emotions at work and medical utilization in Korea

**DOI:** 10.1186/s40557-014-0031-2

**Published:** 2014-10-01

**Authors:** Hongdeok Seok, Jin-Ha Yoon, Wanhyung Lee, June-Hee Lee, Pil Kyun Jung, Inah Kim, Jong-Uk Won, Jaehoon Roh

**Affiliations:** 1 Graduate School of Public Health, Yonsei University, Seoul, Korea; 2 The Institute for Occupational Health, Yonsei University College of Medicine, Seoul, Korea; 3 Department of Preventive Medicine and Public Health, Yonsei University College of Medicine, Seoul, Korea

**Keywords:** Occupational health, Psychological stress, Korea, Health services accessibility, Inpatients, Outpatients, Pharmacy

## Abstract

**Objectives:**

We aimed to investigate the association between concealing emotions at work and medical utilization.

**Methods:**

Data from the 2007–2009 4th Korea National Health and Nutrition Examination Survey (KNHANES IV) was used, 7,094 participants (3,837 males, 3,257 females) aged between 20 and 54 who were economically active and completed all necessary questionnaire items were included. Odds ratios (ORs) and 95% confidence intervals (95% CI) for differences in hospitalization, outpatient visits, and pharmaceutical drug use between those who concealed their emotions and those who did not were investigated using logistic regression models with and without gender stratification.

**Results:**

Among those who concealed their emotions (n = 2,763), 47.4% were females, and 50.1% had chronic disease. In addition, 9.7% of the concealing emotions group had been hospitalized within the last year, 24.8% had been outpatients in the last two weeks, and 28.3% had used pharmaceutical drugs in the last two weeks.

All ORs represent the odds of belonging to the concealing emotions group over the non-concealing emotions group. After adjustment for individual, occupational, socioeconomic and disease factors, the adjusted ORs (95% CI) in hospitalization are 1.29 (1.08 ~ 1.53) in the total population, 1.25 (0.98 ~ 1.60) in males and 1.30 (1.02 ~ 1.66) in females, in outpatient visits are 1.15 (1.02 ~ 1.29) in the total population, 1.05 (0.88 ~ 1.24) in males and 1.25 (1.06 ~ 1.47) in females and in pharmaceutical drug use are 1.12 (1.01 ~ 1.25) in the total population, 1.08 (0.92 ~ 1.27) in males and 1.14 (0.98 ~ 1.33) in females.

**Conclusions:**

Those who concealed their emotions at work were more likely to use medical services. Moreover, the health effects of concealing emotions at work might be more detrimental in women than in men.

## 1 Introduction

Concealing emotions at work is an important requirement of certain types of emotional labor, which leads to occupational stress [1]. Concealing emotions influences the occurrence of various adverse health effects, such as cardiovascular disease and cancer, and also increases mortality [2]. The act of concealing emotions consists of both repression and suppression. Repression refers to the subconscious concealment of emotions, whereas suppression refers to the conscious concealment of emotions [3]. The prevalence of occupational stress related to emotional disturbance such as concealing emotions and its health effects (e.g., burnout) is increasing worldwide [4].

Occupational stress is linked to various chronic and acute diseases, such as cardiovascular disease [5]. Furthermore, occupational stress induces long absences due to sickness [6]. Hence, it is plausible that concealing emotions at work is linked to human health. Although previous studies have investigated the detrimental effects on health from concealing emotions at work, to the best of our knowledge, the association between concealing emotions at work and medical utilization has not yet been investigated.

The investigation of medical utilization is a widely used method for measuring health effects, and can be assessed through several indicators including the number of hospitalizations, outpatient visits, emergency department visits, and frequency of pharmaceutical drug use [7]. The following four subsets of factors have been found to affect medical utilization, namely, individual factors such as gender [8]-[10], age [8],[10], smoking [11], alcohol intake [11],[12], and body mass index (BMI) [7],[11],[13]; occupational factors such as employment status [14] and shift work [15]; socioeconomic factors such as marriage [14], region [16], income [8], education [8], type of public insurance and having additional private insurance [14],[17]-[19]; and individual disease factors such as chronic disease status [8]. Thus, when investigating the effects of concealing emotions at work on increased medical utilization, we should consider controlling for individual, occupational, socioeconomic, and individual disease factors. However, no previous in-depth analyses were found in the literature.

Some reports have suggested that medical utilization differs between men and women [8]-[10]. Thus, a gender-stratified analysis is required to elucidate the association between concealing emotions at work and medical utilization. We aimed to investigate the association between concealing emotions at work and medical utilization using a multivariate logistic regression model that incorporated individual factors, occupational factors, socioeconomic status, and individual disease factors. Furthermore, we conducted a gender-stratified analysis to investigate gender effects.

## 2 Materials and methods

### 2.1 Study design

Data from the 4th Korea National Health and Nutrition Examination Survey (KNHANES IV), collected between 2007 and 2009, were used for all data analyses in this study. Participation in the KNHANES IV was voluntary. All participants provided written informed consent and the Institutional Review Board of the Korea Centers for Disease Control and Prevention approved this study (No. 2007-02-CON-04-P, 2008-04-EXP-01-C, 2009-01-CON-03-2C).

The KNHANES IV was a nationwide cross-sectional study conducted by the Korean Ministry of Health and Welfare. Households were randomly selected for participation using stratified multistage probability sampling based on geographical areas of the Korean population. A total of 600 geographical sampling units were used in the KNHANES IV, which generated a sample of 13,800 households for data collection.

### 2.2 Participants

In total, 24,871 participants from three years of KNHANES IV data (2007–2009) were considered for enrollment in this study (2007 = 4,594, 2008 = 9,744, 2009 = 10,533). Of these, 10,031 economically active participants who completed the questionnaire item on concealing emotions at work were selected. Next, we excluded participants older than 54 and younger than 20, because the average retirement age in Korea is 55 and those younger than 20 were not considered economically active [20]. Thus, 7,094 participants were included, but there were missing values for medical utilization: 6 for hospitalization, 3 for outpatient visits, and 1 for pharmaceutical drug use. Thus, there were 7,088 participants for hospitalization (3,834 males, 3,254 females), 7,091 participants for outpatient visits (3,835 males, 3,256 females), and 7,093 participants for pharmaceutical drug use (3,836 males, 3,257 females) included in the final analyses.

### 2.3 Concealing emotions at work

We used a questionnaire item that asked, “Are you concealing emotions at work?” in order to group participants by whether or not they hide their emotions at work. In response to this question, participants answered 1 = *never*, 2 = *rarely*, 3 = *sometimes*, 4 = *always*. The concealing emotions group included all participants who answered 3 or 4, whereas the non-concealing emotions group included all participants who answered 1 or 2.

### 2.4 Medical utilization

We used three yes/no questionnaire items to assess medical utilization: “Have you been hospitalized in the past year?”, “Have you had an outpatient visits within the last two weeks?”, and “Have you used pharmacy drugs over the last two weeks?”

### 2.5 Covariates

Individual factors were age, smoking, alcohol consumption, and obesity. The smoking group included current smokers, whereas the non-smoking group included those who had never smoked or past smokers. Heavy drinkers were those who consumed an average of ≥7 units of alcohol for men and ≥5 units for women ≥2 days/week. Moderate drinkers included those who consumed more than one glass of alcohol per month over the past year, and non-drinkers included those who never drink or drank less than one glass of alcohol per month over the past year. BMI was categorized into three groups as low weight (BMI < 18.5 kg/m^2^), normal weight (BMI 18.5–25 kg/m^2^), and obese (BMI > 25 kg/m^2^).

Occupational factors included employment status (self-employed or paid worker) and work schedule (shift worker or day worker).

Socioeconomic factors included marital status, region, income, education, type of public insurance and having additional private insurance. Marriage was divided as either married/divorced or never married. Region was dichotomized as urban or rural. Household income was estimated using the quadrants provided by the KNHANES and relabeled as high and low, which includes the top two and bottom two quadrants, respectively. Education was divided among those who only completed middle school or less, those who only graduated from high school or completed some high school, and those who attended college or higher. For classifying public insurance, participants had national health insurance, national medical protection, or none of the above. Additional private insurance was recorded as yes or no.

For disease factors, participants were classified according to chronic disease status (yes or no). We used data on suffering from illness. The KNHANES survey included a question asking “Do you have this disease now?” for the following 40 diseases: hypertension, hyperlipidemia, stroke, myocardial infarction, angina pectoris, hemorrhoids, osteoarthritis, rheumatic arthritis, osteoporosis, lower back pain, pulmonary tuberculosis, non-pulmonary tuberculosis, asthma, chronic obstructive pulmonary disease, sinusitis, bronchiectasis, allergic rhinitis, depression, anemia, atopic dermatitis, chronic renal failure, incontinence, temporomandibular joint disease, diabetic mellitus, thyroid disease, cataract, glaucoma, otitis media, gastric cancer, hepatic cancer, colon cancer, breast cancer, cervical cancer, lung cancer, other cancer group 1, other cancer group 2, gastroduodenal ulcer, chronic hepatitis B, chronic hepatitis C, and liver cirrhosis. We dichotomized chronic disease status, those who answered “Yes” to one or more of the chronic diseases were the chronic disease group, and those who answered “No” to all were the non-chronic disease group.

### 2.6 Statistical analysis

Chi-squared tests were used to identify differences between those who concealed and did not conceal their emotions for general characteristics as well as hospitalization, outpatient visits, and pharmaceutical drug use.

We also conducted a chi-squared test between the hospitalization and non-hospitalization groups, between outpatient and non-outpatient visits groups, and between pharmaceutical drug use and non-pharmaceutical drug use groups in the same way.

We calculated odds ratios (ORs) and 95% confidence intervals (95% CI) for differences in hospitalization, outpatient visits, and pharmaceutical drug use between those who concealed and did not conceal their emotions using logistic regression models for the total population and for men and women separately.

SAS 9.2 (SAS, Inc., Cary, NC, USA) was used for all statistical analyses and a p-value < 0.05 was considered significant.

## 3 Results

### 3.1 Concealing emotions at work

Table 1 presents the differences between groups, in addition to significance values. 2,763 participants were concealing emotions group, whereas 4,331 participants were non-concealing emotions group. In the concealing emotions group, 47.4% were females, 67.7% were 30–49 years old, 18.0% were heavy drinkers, 31.7% were smokers, 31.6% were obese, 65.1% were paid workers, and 22.3% were shift workers, whereas only 17.8% of the non-concealing emotions group were shift workers (p-value < 0.001). Furthermore, 81.6% of the concealing emotions group were married, and 46.3% were urban residents, whereas 43.8% of the non- concealing emotions group were urban residents (p-value = 0.039). Of the concealing emotions group, 48.4% had low income, 81.4% had more than a high school education, 98.3% had national health insurance, 84.4% had additional private insurance, and 50.1% had chronic disease, whereas 43.8% of the non-concealing emotions group had chronic disease (p-value < 0.001).

**Table 1:** Differences between those who concealed or did not conceal their emotions at work

**Variables**		**Concealing emotions at work N(%)**		
		**Yes**	**No**	**P-value**
Gender	Male	1454 (52.6)	2383 (55.0)	0.051
	Female	1309 (47.4)	1948 (45.0)	
Age	20 ~ 29	455 (16.5)	713 (16.5)	0.404
	30 ~ 39	910 (32.9)	1349 (31.1)	
	40 ~ 49	961 (34.8)	1575 (36.4)	
	50 ~ 54	437 (15.8)	694 (16.0)	
Alcohol intake^*^	Heavy drinking	498 (18.0)	787 (18.2)	0.884
	Moderate drinking	1369 (49.6)	2121 (49.0)	
	Non-drinking	893 (32.4)	1420 (32.8)	
Smoking^†^	Current	875 (31.7)	1346 (31.1)	0.612
	Never or past	1885 (68.3)	2982 (68.9)	
BMI^‡^	Low	123 ( 4.5)	187 ( 4.4)	0.816
	Normal	1756 (63.9)	2716 (63.3)	
	Obese	869 (31.6)	1387 (32.3)	
Work type	Paid worker	1797 (65.1)	2883 (66.6)	0.199
	Self-employed	964 (34.9)	1446 (33.4)	
Work schedule	Shift worker	616 (22.3)	769 (17.8)	<0.001
	Day worker	2141 (77.7)	3552 (82.2)	
Married	Yes	2243 (81.6)	3491 (81.2)	0.700
	No	507 (18.4)	810 (18.8)	
Region	Urban	1280 (46.3)	1897 (43.8)	0.039
	Rural	1483 (53.7)	2434 (56.2)	
Income^§^	High	1406 (51.6)	2224 (52.1)	0.675
	Low	1320 (48.4)	2043 (47.9)	
Education^||^	Middle school or lower	513 (18.6)	774 (17.9)	0.736
	High school	1167 (42.2)	1857 (42.9)	
	College or higher	1083 (39.2)	1699 (39.2)	
Public insurance	National health insurance	2703 (98.2)	4233 (98.4)	0.084
	National medical protection	43 ( 1.6)	68 ( 1.6)	
	Nothing	5 ( 0.2)	1 ( 0.0)	
Additional private insurance	Yes	2284 (84.4)	3567 (84.3)	0.967
	No	423 (15.6)	664 (15.7)	
Chronic Disease	Yes	1384 (50.1)	1896 (43.8)	<0.001
	No	1379 (49.9)	2435 (56.2)	
Hospitalization	Yes	267 ( 9.7)	333 ( 7.7)	0.004
	No	2494 (90.3)	3994 (92.3)	
Outpatients visits	Yes	684 (24.8)	953 (22.0)	0.008
	No	2077 (75.2)	3377 (78.0)	
Pharmaceutical drug use	Yes	782 (28.3)	1100 (25.4)	0.008
	No	1981 (71.7)	3230 (74.6)	

^*^Heavy drinkers were those who consumed an average of ≥7 units of alcohol for men and ≥5 units for women ≥ 2 days/week. Moderate drinkers included those who consumed more than one glass of alcohol per month over the past year, and non-drinkers included those who never drink or drank less than one glass of alcohol per month over the past year.

^†^Current smokers or never/past smokers.

^‡^Body mass index (BMI) was categorized into three groups as low weight (BMI < 18.5 kg/m^2^), normal weight (BMI 18.5–25 kg/m^2^), and obese (BMI > 25 kg/m^2^).

^§^Household income was estimated using the quadrants provided by the KNHANES and relabeled as high and low, which includes the top two an bottom two quadrants, respectively.

^||^Education was divided among those who only completed middle school or less, those who only graduated from high school or completed some high school, and those who attended college or higher.

Regarding medical utilization, 9.7% of the concealing emotions group and 7.7% of the non-concealing emotions group had been hospitalized within the last year (p-value = 0.004), 24.8% of the concealing emotions group and 22.0% of the non-concealing emotions group had been outpatients within the last two weeks (p-value = 0.008), and 28.3% of concealing emotions group and 25.4% of the non-concealing emotions group had used pharmaceutical drugs in the last two weeks (p-value = 0.008).

### 3.2 Medical utilization

Table 2 presents all differences and significance values between hospitalization groups. 600 participants were hospitalization group, whereas 6,488 participants were non-hospitalization group. The hospitalization group included 9.5% of females and 7.6% of males respondents (p-value = 0.006), 7.8% of heavy drinkers, 7.3% of moderate drinkers and 10.6% of non-drinkers (p-value < 0.001), 7.4% of smokers and 8.9% of non-smokers (p-value = 0.041), 8.9% of married people and 6.9% of non-married people (p-value = 0.024), and 10.2% of the lowest education group—a higher proportion than the other education groups (p-value = 0.040). The hospitalization group also included 9.6% of those with chronic disease and 7.5% of those without chronic disease (p-value = 0.002).

**Table 2:** Variable differences by hospitalization, outpatient visits and pharmaceutical drug use

**Variables**		**Hospitalization N(%)**			**Outpatients visits N(%)**			**Pharmaceutical drug use N(%)**		
		**Yes**	**No**	**P-value**	**Yes**	**No**	**P-value**	**Yes**	**No**	**P-value**
Gender	Male	292 ( 7.6)	3542 (92.4)	0.006	737 (19.2)	3098 (80.8)	<0.001	863 (22.5)	2973 (77.5)	<0.001
	Female	308 ( 9.5)	2946 (90.5)		900 (27.6)	2356 (72.4)		1019 (31.3)	2238 (68.7)	
Age	20 ~ 29	107 ( 9.2)	1061 (90.8)	0.090	250 (21.4)	916 (78.6)	<0.001	329 (28.2)	839 (71.8)	0.001
	30 ~ 39	196 ( 8.7)	2062 (91.3)		439 (19.4)	1820 (80.6)		535 (23.7)	1724 (76.3)	
	40 ~ 49	188 ( 7.4)	2345 (92.6)		598 (23.6)	1937 (76.4)		680 (26.8)	1855 (73.2)	
	50 ~ 54	109 ( 9.7)	1020 (90.3)		350 (30.9)	781 (69.1)		338 (29.9)	793 (70.1)	
Alcohol intake^*^	Heavy drinking	100 ( 7.8)	1184 (92.2)	<0.001	245 (19.1)	1040 (80.9)	<0.001	308 (24.0)	977 (76.0)	0.001
	Moderate drinking	255 ( 7.3)	3233 (92.7)		776 (22.2)	2712 (77.8)		899 (25.8)	2591 (74.2)	
	Non-drinking	245 (10.6)	2065 (89.4)		614 (26.6)	1698 (73.4)		673 (29.1)	1639 (70.9)	
Smoking^†^	Current	165 ( 7.4)	2054 (92.6)	0.041	390 (17.6)	1829 (82.4)	<0.001	495 (22.3)	1726 (77.7)	<0.001
	Never or past	434 ( 8.9)	4429 (91.1)		1245 (25.6)	3621 (74.4)		1385 (28.5)	3481 (71.5)	
BMI^‡^	Low	23 ( 7.4)	287 (92.6)	0.485	74 (23.9)	236 (76.1)	0.082	92 (29.7)	218 (70.3)	0.341
	Normal	365 ( 8.2)	4101 (91.8)		992 (22.2)	3477 (77.8)		1171 (26.2)	3300 (73.8)	
	Obese	201 ( 8.9)	2055 (91.1)		555 (24.6)	1701 (75.4)		611 (27.1)	1645 (72.9)	
Work type	Paid worker	386 ( 8.3)	4292 (91.7)	0.381	1045 (22.3)	3635 (77.7)	0.038	1218 (26.0)	3462 (74.0)	0.174
	Self-employed	214 ( 8.9)	2192 (91.1)		591 (24.6)	1816 (75.4)		664 (27.6)	1745 (72.4)	
Work schedule	Shift worker	116 ( 8.4)	1268 (91.6)	0.971	300 (21.7)	1083 (78.3)	0.174	361 (26.1)	1023 (73.9)	0.686
	Day worker	481 ( 8.5)	5207 (91.5)		1335 (23.5)	4357 (76.5)		1518 (26.7)	4175 (73.3)	
Married	Yes	509 ( 8.9)	5220 (91.1)	0.024	1349 (23.5)	4383 (76.5)	0.058	1507 (26.3)	4226 (73.7)	0.362
	No	91 ( 6.9)	1225 (93.1)		277 (21.0)	1039 (79.0)		363 (27.6)	954 (72.4)	
Region	Urban	250 ( 7.9)	2925 (92.1)	0.117	737 (23.2)	2440 (76.8)	0.862	876 (27.6)	2300 (72.4)	0.076
	Rural	350 ( 8.9)	3563 (91.1)		900 (23.0)	3014 (77.0)		1006 (25.7)	2911 (74.3)	
Income^§^	High	317 ( 8.7)	3310 (91.3)	0.513	842 (23.2)	2788 (76.8)	0.990	916 (25.2)	2714 (74.8)	0.009
	Low	278 ( 8.3)	3082 (91.7)		778 (23.2)	2582 (76.8)		942 (28.0)	2420 (72.0)	
Education^||^	Middle school or lower	131 (10.2)	1154 (89.8)	0.040	388 (30.1)	899 (69.9)	<0.001	402 (31.2)	885 (68.8)	<0.001
	High school	238 ( 7.9)	2783 (92.1)		645 (21.3)	2377 (78.7)		790 (26.1)	2233 (73.9)	
	College or higher	230 ( 8.3)	2551 (91.7)		604 (21.7)	2177 (78.3)		690 (24.8)	2092 (75.2)	
Public insurance	National health insurance	589 ( 8.5)	6341 (91.5)	0.658	1587 (22.9)	5346 (77.1)	0.018	1830 (26.4)	5105 (73.6)	0.111
	National medical protection	11 ( 9.9)	100 (90.1)		38 (34.2)	73 (65.8)		37 (33.3)	74 (66.7)	
	Nothing	0 ( 0.0)	6 (100.0)		1 (16.7)	5 (83.3)		3 (50.0)	3 (50.0)	
Additional private insurance	Yes	511 ( 8.7)	5335 (91.3)	0.153	1376 (23.5)	4474 (76.5)	0.048	1566 (26.8)	4284 (73.2)	0.399
	No	80 ( 7.4)	1006 (92.6)		225 (20.7)	861 (79.3)		277 (25.5)	810 (74.5)	
Chronic disease	Yes	314 ( 9.6)	2962 (90.4)	0.002	976 (29.8)	2303 (70.2)	<0.001	1060 (32.3)	2219 (67.7)	<0.001
	No	286 ( 7.5)	3526 (92.5)		661 (17.3)	3151 (82.7)		822 (21.6)	2992 (78.4)	

^*^Heavy drinkers were those who consumed an average of ≥7 units of alcohol for men and ≥5 units for women ≥ 2 days/week. Moderate drinkers included those who consumed more than one glass of alcohol per month over the past year, and non-drinkers included those who never drink or drank less than one glass of alcohol per month over the past year.

^†^Current smokers or never/past smokers.

^‡^Body mass index (BMI) was categorized into three groups as low weight (BMI < 18.5 kg/m^2^), normal weight (BMI 18.5–25 kg/m^2^), and obese (BMI > 25 kg/m^2^).

^§^Household income was estimated using the quadrants provided by the KNHANES and relabeled as high and low, which includes the top two an bottom two quadrants, respectively.

^||^Education was divided among those who only completed middle school or less, those who only graduated from high school or completed some high school, and those who attended college or higher.

Table 2 also presents all differences and significance values for the outpatient visits groups. 1,637 participants were outpatient visits group, whereas 5,454 participants were non-outpatient visits group. The outpatient visits group included 27.6% of females and 19.2% of males respondents (p-value < 0.001), 30.9% of participants over 50 years old (the highest of all age groups) (p-value < 0.001), 19.1% of heavy drinkers, 22.2% of moderate drinkers and 26.6% of non-drinkers (p-value < 0.001), 17.6% of smokers and 25.6% of non-smokers (p-value < 0.001), 22.3% of paid workers and 24.6% of self-employees (p-value = 0.038), and 30.1% of the lowest education group—the highest among all education groups (p-value < 0.001). This group also included 34.2% of participants with national medical protection (the highest of all public insurance groups) (p-value = 0.018), 23.5% of those with additional private insurance and 20.7% those without additional private insurance, and 29.8% of those with chronic disease, but only 17.3% of those without chronic disease (p-value < 0.001).

Table 2 also presents all differences between pharmaceutical drug use groups, along with significance values. 1,882 participants were pharmaceutical drug use group, whereas 5,211 participants were non-pharmaceutical drug use group. The pharmaceutical drug use group included 31.3% of females but only 22.5% of males respondents (p-value < 0.001), 29.9% of participants over 50 (the highest of all age groups) (p-value = 0.001), 24.0% of heavy drinkers, 25.8% of moderate drinkers and 29.1% of non-drinkers (p-value = 0.001), 22.3% of smokers and 28.5% of non-smokers (p-value < 0.001), 28.0% of participants with a low income but only 25.2% of those with a high income (p-value = 0.009), 31.2% of participants in the lowest education group (the highest of all education groups) (p-value < 0.001), and 32.3% of those with a chronic disease, but only 21.6% of those without a chronic disease (p-value < 0.001).

### 3.3 Association between concealing emotions at work and medical utilization

All ORs represent the odds of belonging to the concealing emotions group over the non-concealing emotions group. The crude ORs (95% CI) were 1.28 (1.09–1.52) for hospitalization, 1.17 (1.04–1.31) for outpatient visits, and 1.16 (1.04–1.29) for pharmaceutical drug use. In Model I, adjusting for gender, age, alcohol consumption, smoking, and obesity, the adjusted ORs (95% CI) were 1.30 (1.09–1.54) for hospitalization, 1.17 (1.04–1.31) for outpatient visits, and 1.15 (1.03–1.28) for pharmaceutical drug use. In Model II, further adjusting for employment status, work schedule, marriage, region, income, education, type of public insurance, and additional private insurance, the adjusted ORs (95% CI) were 1.30 (1.10–1.55) for hospitalization, 1.19 (1.06–1.33) for outpatient visits, and 1.15 (1.03–1.29) for pharmaceutical drug use. In Model III, further adjusting for individual chronic disease, the adjusted ORs (95% CI) were 1.29 (1.08–1.53) for hospitalization, 1.15 (1.02–1.29) for outpatient visits, and 1.12 (1.01–1.25) for pharmaceutical drug use.

We then conducted advanced logistic regression models with gender stratification. All odds ratios again represent the odds of belonging to the concealing emotions group over the non-concealing emotions group. For males, the crude ORs (95% CI) were 1.23 (0.97–1.57) for hospitalization, 1.05 (0.89–1.24) for outpatient visits, and 1.10 (0.94–1.28) for pharmaceutical drug use. In Model I, for males, the adjusted ORs (95% CI) were 1.26 (0.99–1.61) for hospitalization, 1.06 (0.89–1.25) for outpatient visits, and 1.10 (0.94–1.28) for pharmaceutical drug use. In Model II, for males, the adjusted ORs (95% CI) were 1.27 (0.99–1.62) for hospitalization, 1.07 (0.91–1.27) for outpatient visits, and 1.11 (0.94–1.30) for pharmaceutical drug use. In Model III, for males, the adjusted ORs (95% CI) were 1.25 (0.98–1.60) for hospitalization, 1.05 (0.88–1.24) for outpatient visits, and 1.08 (0.92–1.27) for pharmaceutical drug use.

Next, we conducted identical advanced logistic regression models for females. The crude ORs (95% CI) were 1.33 (1.05–1.68) for hospitalization, 1.26 (1.08–1.47) for outpatient visits, and 1.20 (1.03–1.39) for pharmaceutical drug use. In Model I, for females, the adjusted ORs (95% CI) were 1.34 (1.05–1.70) for hospitalization, 1.27 (1.09–1.49) for outpatient visits, and 1.20 (1.03–1.39) for pharmaceutical drug use. In Model II, for females, the adjusted ORs (95% CI) were 1.32 (1.03–1.69) for hospitalization, 1.30 (1.11–1.53) for outpatient visits, and 1.18 (1.01–1.38) for pharmaceutical drug use. In Model III, for females, the adjusted ORs (95% CI) were 1.30 (1.02–1.66) for hospitalization, 1.25 (1.06–1.47) for outpatient visits, and 1.14 (0.98–1.33) for pharmaceutical drug use (Table 3).

**Table 3:** Results of the logistic regression analyses

**Variables**	**Crude**	Model I^*^	**Model II**^**†**^	**Model III**^**‡**^
Total population				
Hospitalization	1.28 (1.09 ~ 1.52)	1.30 (1.09 ~ 1.54)	1.30 (1.10 ~ 1.55)	1.29 (1.08 ~ 1.53)
Outpatients visits	1.17 (1.04 ~ 1.31)	1.17 (1.04 ~ 1.31)	1.19 (1.06 ~ 1.33)	1.15 (1.02 ~ 1.29)
Pharmaceutical drug use	1.16 (1.04 ~ 1.29)	1.15 (1.03 ~ 1.28)	1.15 (1.03 ~ 1.29)	1.12 (1.01 ~ 1.25)
Males				
Hospitalization	1.23 (0.97 ~ 1.57)	1.26 (0.99 ~ 1.61)	1.27 (0.99 ~ 1.62)	1.25 (0.98 ~ 1.60)
Outpatients visits	1.05 (0.89 ~ 1.24)	1.06 (0.89 ~ 1.25)	1.07 (0.91 ~ 1.27)	1.05 (0.88 ~ 1.24)
Pharmaceutical drug use	1.10 (0.94 ~ 1.28)	1.10 (0.94 ~ 1.28)	1.11 (0.94 ~ 1.30)	1.08 (0.92 ~ 1.27)
Females				
Hospitalization	1.33 (1.05 ~ 1.68)	1.34 (1.05 ~ 1.70)	1.32 (1.03 ~ 1.69)	1.30 (1.02 ~ 1.66)
Outpatients visits	1.26 (1.08 ~ 1.47)	1.27 (1.09 ~ 1.49)	1.30 (1.11 ~ 1.53)	1.25 (1.06 ~ 1.47)
Pharmaceutical drug use	1.20 (1.03 ~ 1.39)	1.20 (1.03 ~ 1.39)	1.18 (1.01 ~ 1.38)	1.14 (0.98 ~ 1.33)

^*^Model I: Adjusted for individual factors (gender, age, alcohol intake, smoking, BMI).

^†^Model II: Model I + Adjusted for occupational factors (employment status, work schedule) and socioeconomic factors (marriage, region, income, education, type of public insurance, additional private insurance).

^‡^Model III: Model II + Adjusted for individual disease factor (chronic disease).

## 4 Discussion

Our current study shows that the concealing emotions group had a higher rate of hospitalization, outpatient visits, and pharmaceutical drug use. This finding was consistent across all models before gender stratification. This finding suggests that concealing emotions is detrimental for health as measured by increased medical utilization. Concealing emotions is a kind of occupational stress, which is associated with many acute and chronic diseases [1],[5]. Moreover, occupational stress is related to depression which is closely related to many other acute and chronic diseases [21],[22]. Concealing emotions group has a higher rate of medical utilization due to these reasons. For males, compared to the non-concealing emotions group, the concealing emotions group had a higher rate of hospitalization, outpatient visits, and pharmaceutical drug use, but this result was non-significant across all models. Conversely, in females, the concealing emotions group has higher rates of hospitalization, outpatient visits, and pharmaceutical drug use. All adjusted models, except Model III, were statistically significant. Therefore, concealing emotions at work might be more detrimental for women than it is for men, especially since gender is an important factor in determining medical utilization [8]-[10]. Furthermore, concealing emotions at work might causes more occupational stress among females than males [23],[24]. Previous studies have reported that females experience more stress than males do because of differences in perception, exposure to work, physical capacity, and burdens having to do with their household [25]-[29].

The concealing emotions group had a greater proportion of shift workers than the non-concealing group. Shift workers tend to work as call center operators, health care providers, and nurses, all of whom require concealing emotions by the nature of their work [30]. Because both concealing emotions and shift work are detrimental to health, the implications of these two factors is of concern [15]. The concealing emotions group also tended to be urban residents to a greater extent than the non-concealing emotions group. This finding might suggest that concealing emotions is required in office jobs, which is predominant in urban environments. The concealing emotions group also had a higher rate of chronic disease, hospitalization, outpatient visits, and pharmaceutical drug use, suggesting that concealing emotions at work is harmful to health. This finding supports the finding that concealing emotions is harmful to health in general [2]. This might have resulted because emotional experiences have been found to affect physiologic health such as the immune system [31].

According to all medical utilization indexes, females tend to access medical services more than males, suggesting that females are more active in medical utilization, which has been supported by previous studies [8]-[10]. Since healthy individuals are more likely to drink alcohol and smoke cigarettes (Similar to sick person effects) [32], and Korean males drink and smoke more often than females [33],[34], in contrary to our expectations, alcohol drinkers and cigarette smokers in our study used medical services less than those who did not drink alcohol or smoke cigarettes. The rate of self-employment among those who visited an outpatient clinic was higher than that among paid workers. One possible explanation for this result is that clinic operating hours and working hours overlap. In low income group, the rate of pharmaceutical drug use was higher than those in high income group was probably due to their limited access to hospitalization and outpatients visits. However, this result is not consistent with previous research [35]. The lowest education group in our study used more medical services since older people had lower education than younger people in Korea and this result was also supported by previous studies [36],[37]. The national medical protection group and having private insurance group visited the outpatient clinic more frequently than other groups, and this could be induced by moral hazard [18],[19],[38].

Emotional labor is defined as work in which emotional demands are inherent. Employees are expected to express certain emotions to customers to meet the demands of their job and they can be reprimanded when they do not meet the emotional demands [39]. Emotional labor is one component of job stress [40] and is related to various chronic diseases such as cardiovascular disease [2]. Concealing emotions at work is a crucial component of emotional labor. Thus, in accordance with our findings, we cautiously suggest that female workers in fields that require emotional concealment tend to use more medical services. Currently, emotional labor is a controversial issue in Korean labor policy. Workers have been forced to conceal their emotions at work. However, few policies exist that protect workers who have to conceal their emotions at work. Moreover, few studies have investigated the health effects of concealing emotions at work in Korea. We hope that this study will play the role of cornerstone in the establishment of future labor policies in Korea that deal with concealing emotions at work.

Our study had some clear strengths. First, we included a large sample size with more than 7,000 participants. Second, we investigated the association between concealing emotions at work and medical utilization with and without gender stratification. Third, potential confounders such as individual factors (model I), occupation and socioeconomic factors (model II), and individual diseases factor (model III) were adjusted for in our statistical analyses. Confounder adjustment increased the level of C-values and reduced the level of Akaike Information Criterion (AIC). For example, C-value increased from 0.534 to 0.623 and AIC decreased from 2037 to 1908 for hospitalization among females. Fourth, to the best of our knowledge, this was the first study investigating the association between concealing emotions at work and medical utilization.

Our study also had some limitations. First, the main independent variable—concealing emotions at work— was based on a subjective answer to only one questionnaire item. Thus, we were not able to objectively determine whether participants did or did not conceal their emotions at work, which might lead to a non-differential misclassification and underestimation. Second, medical utilization was measured using a subjective questionnaire. Many other methods for acquiring information on medical utilization exist, such as checking insurance data and checking hospital data. In some cases, information obtained by self-report methods does not correspond with information obtained by checking insurance data or hospital data due to potential biases, such as recall bias. However, in Korea, insurance and hospital data are protected by Korean law. Thus, self-report is considered the next best method and has been used in many studies [41],[42]. Nevertheless, we believe that our large sample size might have outweighed the potential benefits of using a different method of data collection. Third, the calculated C-values of our models were low (0.580 ~ 0.629). Thus, our logistic regression models might not have been able to provide enough explanation for the influence of concealing emotions on medical utilization. Further studies are needed.

In summary, we found that those who concealed emotions at work were more likely to use medical services, with higher rates of hospitalization, outpatient visits, and pharmaceutical drug use than those who did not conceal emotions were. When we stratified the data by gender, no statistical significance among males was found, but a significant difference was found among females. Thus, the health effects of concealing emotions at work might be more detrimental for women than it is for men. We anticipate that our study, with over 7,000 participants, provides important insights into the health consequences of concealing emotions at work.

## Competing interests

The authors declare that they have no competing interests.

## Authors’ contributions

HS: The first author of this article. He designed the study, collected and interpreted the data, and drafted the manuscript. JHY: He suggested the study design, interpreted the data, and revised the manuscript. WL: He suggested the study design, interpreted the data, and revised the manuscript. JHL: He collected the data and drafted the manuscript. PKJ: He collected the data and drafted the manuscript. IK: She suggested the study design, interpreted the data, and revised the manuscript. JUW: He suggested the study design, interpreted the data, and revised the manuscript. JR: The corresponding author of this article. He suggested the study design, interpreted the data, and revised the manuscript. All authors have approved the final version of the manuscript.

## References

[B1] Cheng C Bartram T Karimi L Leggat SG The role of team climate in the management of emotional labour: implications for nurse retention J Adv Nurs 2013 69 12 2812 2825 10.1111/jan.12202 23834619

[B2] Chapman BP Fiscella K Kawachi I Duberstein P Muennig P Emotion suppression and mortality risk over a 12-year follow-up J Psychosom Res 2013 75 4 381 385 10.1016/j.jpsychores.2013.07.014 24119947 PMC3939772

[B3] Myers LB Vetere A Derakshan N Are suppression and repressive coping related? Pers Indiv Differ 2004 36 5 1009 1013 10.1016/S0191-8869(03)00196-X

[B4] de Castro AB Agnew J Fitzgerald ST Emotional labor: relevant theory for occupational health practice in post-industrial America AAOHN J 2004 52 3 109 115 15068101

[B5] De Bacquer D Pelfrene E Clays E Mak R Moreau M de Smet P Kornitzer M De Backer G Perceived job stress and incidence of coronary events: 3-year follow-up of the Belgian Job Stress Project cohort Am J Epidemiol 2005 161 5 434 441 10.1093/aje/kwi040 15718479

[B6] Slany C Schutte S Chastang JF Parent-Thirion A Vermeylen G Niedhammer I Psychosocial work factors and long sickness absence in Europe Int J Occup Environ Health 2014 20 1 16 25 10.1179/2049396713Y.0000000048 24176393 PMC4137803

[B7] Goetzel RZ Gibson TB Short ME Chu BC Waddell J Bowen J Lemon SC Fernandez ID Ozminkowski RJ Wilson MG DeJoy DM A multi-worksite analysis of the relationships among body mass index, medical utilization, and worker productivity J Occup Environ Med 2010 52 Suppl 1 S52 S58 10.1097/JOM.0b013e3181c95b84 20061888 PMC2862260

[B8] Vikum E Krokstad S Westin S Socioeconomic inequalities in health care utilisation in Norway: the population-based HUNT3 survey Int J Equity Health 2012 11 48 10.1186/1475-9276-11-48 22909009 PMC3490903

[B9] Cameron KA Song J Manheim LM Dunlop DD Gender disparities in health and healthcare use among older adults J Women’s Health 2010 19 9 1643 1650 10.1089/jwh.2009.1701 PMC2965695 20695815

[B10] Dunlop DD Manheim LM Song J Chang RW Gender and ethnic/racial disparities in health care utilization among older adults J Gerontol Ser B Psychol Sci Soc Sci 2002 57 4 S221 S233 10.1093/geronb/57.4.S221 12084792

[B11] Vals K Kiivet RA Leinsalu M Alcohol consumption, smoking and overweight as a burden for health care services utilization: a cross-sectional study in Estonia BMC Public Health 2013 13 772 10.1186/1471-2458-13-772 23968192 PMC3765337

[B12] Risal A Tharoor H A cross-sectional comparison of drinking patterns, alcohol use and related medical morbidities in a secondary versus tertiary setting Kathmandu Univ Med J (KUMJ) 2013 11 42 152 157 24096224 10.3126/kumj.v11i2.12492

[B13] Quesenberry CP Jr Caan B Jacobson A Obesity, health services use, and health care costs among members of a health maintenance organization Arch Intern Med 1998 158 5 466 472 10.1001/archinte.158.5.466 9508224

[B14] Meer J Rosen HS Insurance and the utilization of medical services Soc Sci Med 2004 58 9 1623 1632 10.1016/S0277-9536(03)00394-0 14990364

[B15] Bambra CL Whitehead MM Sowden AJ Akers J Petticrew MP Shifting schedules: the health effects of reorganizing shift work Am J Prev Med 2008 34 5 427 434 10.1016/j.amepre.2007.12.023 18407011

[B16] Krumkamp R Sarpong N Kreuels B Ehlkes L Loag W Schwarz NG Zeeb H Adu-Sarkodie Y May J Health care utilization and symptom severity in Ghanaian children–a cross-sectional study PLoS One 2013 8 11 e80598 10.1371/journal.pone.0080598 24244698 PMC3828249

[B17] Jerant A Fiscella K Tancredi DJ Franks P Health insurance is associated with preventive care but not personal health behaviors J Am Board Fam Med 2013 26 6 759 767 10.3122/jabfm.2013.06.130054 24204073

[B18] Jeon B Kwon S Effect of private health insurance on health care utilization in a universal public insurance system: a case of South Korea Health Policy 2013 113 1–2 69 76 10.1016/j.healthpol.2013.05.007 23786992

[B19] Kang S You CH Kwon YD Oh EH Effects of supplementary private health insurance on physician visits in Korea J Formos Med Assoc 2009 108 12 912 920 10.1016/S0929-6646(10)60003-4 20040455

[B20] Jin-Ha Yoon K-HL Kyu-Yeon H Sei Jin C Bong Suk C Seong Ho M Kyung Suk Lee H-SC Aeyong E Sang Baek K Suicide trend of standardized mortality ratio and Age standardized proportion mortality ratio according to occupational groups in Korea: 1993–2007 Korean J Occup Environ Med 2011 23 2 173 182

[B21] Deguchi Y Inoue K Muramatsu T Iwasaki S Yamauchi T Nakao T Muroya M Kobayashi Y Kato Y Kiriike N Relationships between occupational stress and depressive symptoms among prison officers in Japan Osaka City Med J 2013 59 2 91 98 24575584

[B22] Egede LE Depression: greater effect on overall health than angina, arthritis, asthma or diabetes Evid Based Ment Health 2008 11 2 57 10.1136/ebmh.11.2.57 18441146

[B23] Johnson HA Spector PE Service with a smile: do emotional intelligence, gender, and autonomy moderate the emotional labor process? J Occup Health Psychol 2007 12 4 319 333 10.1037/1076-8998.12.4.319 17953492

[B24] Yu SF Gu GZ Zhou WH Zhou SY Yang XF Sun SY Gender difference of relationship between occupational stress and depressive symptoms Zhonghua Lao Dong Wei Sheng Zhi Ye Bing Za Zhi 2011 29 12 887 892 22357526

[B25] Herrero SG Saldana MA Rodriguez JG Ritzel DO Influence of task demands on occupational stress: gender differences J Saf Res 2012 43 5–6 365 374 10.1016/j.jsr.2012.10.005 23206509

[B26] Hooftman WE van der Beek AJ Bongers PM van Mechelen W Gender differences in self-reported physical and psychosocial exposures in jobs with both female and male workers J Occup Environ Med 2005 47 3 244 252 10.1097/01.jom.0000150387.14885.6b 15761320

[B27] Nordander C Ohlsson K Balogh I Hansson GA Axmon A Persson R Skerfving S Gender differences in workers with identical repetitive industrial tasks: exposure and musculoskeletal disorders Int Arch Occup Environ Health 2008 81 8 939 947 10.1007/s00420-007-0286-9 18066574

[B28] Matthews S Hertzman C Ostry A Power C Gender, work roles and psychosocial work characteristics as determinants of health Soc Sci Med 1998 46 11 1417 1424 10.1016/S0277-9536(97)10141-1 9665571

[B29] Sekine M Chandola T Martikainen P Marmot M Kagamimori S Sex differences in physical and mental functioning of Japanese civil servants: explanations from work and family characteristics Soc Sci Med 2010 71 12 2091 2099 10.1016/j.socscimed.2010.09.031 21041011

[B30] Bagdasarov Z Connelly S Emotional labor among healthcare professionals: the effects are undeniable Narrative Inq Bioeth 2013 3 2 125 129 10.1353/nib.2013.0040 24407084

[B31] Salovey P Rothman AJ Detweiler JB Steward WT Emotional states and physical health Am Psychol 2000 55 1 110 121 10.1037/0003-066X.55.1.110 11392855

[B32] Sterling TD Weinkam JJ Weinkam JL The sick person effect J Clin Epidemiol 1990 43 2 141 151 10.1016/0895-4356(90)90177-Q 2303844

[B33] http://kosis.kr/ups3/service/ch_file_down.jsp?PUBCODE=KP&FILE_NAME=%2Fups3%2Fupload%2F101%2FKP0207.xls&SEQ=3080&_ORG_ID=101 Service. KKSI: **The drinking alcohol rate in Korea.** . Available: [cited 6 Oct 2014].

[B34] http://kosis.kr/ups3/service/ch_file_down.jsp?PUBCODE=KP&FILE_NAME=%2Fups3%2Fupload%2F101%2FKP0207.xls&SEQ=3080&_ORG_ID=101 Service. KKSI: **The smoking rate in Korea.**ᅟ. Available: [cited 6 Oct 2014].

[B35] Lipworth L Mumma MT Cavanaugh KL Edwards TL Ikizler TA Tarone RE McLaughlin JK Blot WJ Incidence and predictors of end stage renal disease among low-income blacks and whites PLoS One 2012 7 10 e48407 10.1371/journal.pone.0048407 23110237 PMC3480508

[B36] Van der Heyden JH Demarest S Tafforeau J Van Oyen H Socio-economic differences in the utilisation of health services in Belgium Health Policy 2003 65 2 153 165 10.1016/S0168-8510(02)00213-0 12849914

[B37] http://kosis.kr/ups3/service/ch_file_down.jsp?PUBCODE=KP&FILE_NAME=%2Fups3%2Fupload%2F101%2FKP0410.xls&SEQ=3126&_ORG_ID=101 Service. KKSI: **Social indicators in Korea.** 2012, Available: [cited 6 Oct 2014].

[B38] Kiil A What characterises the privately insured in universal health care systems? A review of the empirical evidence Health Policy 2012 106 1 60 75 10.1016/j.healthpol.2012.02.019 22459052

[B39] Brotheridge CM Grandey AA Emotional labor and burnout: comparing two perspectives of “people work” J Vocat Behav 2002 60 1 17 39 10.1006/jvbe.2001.1815

[B40] Katayama H Relationship between emotional labor and job-related stress among hospital nurses Nippon Eiseigaku Zasshi 2010 65 4 524 529 10.1265/jjh.65.524 20885079

[B41] Reichert AR, Augurzky B, Tauchmann H: **Self-perceived job insecurity and the demand for medical rehabilitation: does fear of unemployment reduce health care utilization?** *Health Econ* 2013, doi10.1002/hec.2995. 10.1002/hec.2995 24123554

[B42] Peersman W Pasteels I Cambier D De Maeseneer J Willems S Validity of self-reported utilization of physician services: a population study Eur J Pub Health 2014 24 1 91 97 10.1093/eurpub/ckt079 23813707

